# Emerging trends and focus for the link between the gastrointestinal microbiome and kidney disease

**DOI:** 10.3389/fcimb.2022.946138

**Published:** 2022-08-15

**Authors:** Jiaxing Tan, Ming Chen, Yutong Wang, Yi Tang, Wei Qin

**Affiliations:** ^1^ Division of Nephrology, Department of Medicine, West China Hospital, Sichuan University, Chengdu, China; ^2^ West China School of Medicine, Sichuan University, Chengdu, China

**Keywords:** kidney disease, gastrointestinal microbiome, bibliometric, research focus, probiotics

## Abstract

The clinical value of the relationship between gastrointestinal microbiome imbalance and its corresponding interventions with kidney disease is emerging. This study describes the hotspots and evolution of gastrointestinal microbiome and kidney disease research over the past three decades by scientific collaboration networks and finally predicts future trends in the field through bibliometric analysis and visualization studies. CiteSpace was used to explore the original articles from January 1990 to August 2021 to visualize the knowledge network of journals, countries, institutions, authors, references, and keywords in this field. Publications were extracted from Web of Science Core Collection database using the terms “gastrointestinal microbiome” and “kidney disease” (and their synonyms in MeSH). A total of 2145 publications with 93880 references in 102 journals were included in the analyses. The number of studies combining gastrointestinal microbiomes with kidney diseases has increased significantly over the past two decades. The United States is the leading country in the number of documents, and the leading institution is the Cleveland Clinic. The most landmark articles in the field are on chronic renal failure, L-Carnitin, and cardiovascular disease. The pathogenesis of uremia toxin is an emerging trend in gastrointestinal microbiomes and kidney diseases. In addition, probiotic or synbiotic supplements have strong clinical value in adjusting abnormal intestinal symbiotic environments. This study demonstrates a growing understanding of the interaction between gut microbiota and kidney disease over time. Using microbial supplements to improve the living conditions of kidney disease patients is a promising and hot research focus. Based on publications extracted from the database, this study may provide clinicians and researchers with valuable information to identify potential collaborators and partner institutions and better predict their dynamic progression.

## Introduction

Chronic kidney disease (CKD) has become one of the chronic diseases with the heaviest economic burden globally, and the number of CKD patients is increasing rapidly (Luyckx et al., 2018). According to inductive statistics, the global prevalence of chronic kidney disease fluctuates between 8% and 16%, causing progressed renal dysfunction and metabolic-related complications (Jha et al., 2013; Magliocca et al., 2022). However, its pathogenesis remains unclear, and there is currently no specific treatment for CKD. In the past decade, with the upgrading of research methods, researchers have gradually shifted their focus to the gut to determine the pathogenesis of CKD (Yang et al., 2018). Many scholars have put forward the theory of the “gut-kidney axis”, arguing that there is a close relationship between intestinal flora, intestinal mucosal immunity, and kidneys (Ikee et al., 2020; Wang et al., 2020; Yin et al., 2021).

The human gastrointestinal tract is home to tens of thousands of microbial communities, mainly composed of bacteria (Lavelle and Sokol, 2020). There are intrinsic links between the changes of intestinal flora and kidney pathophysiology (Linh et al., 2022). Unbalanced intestinal flora may lead to abnormal intestinal metabolism and produce more uremic toxins, such as p-cresol sulfate, trimethylamine-N-oxide, and indole sulfate, which may accelerate the occurrence and development of chronic kidney disease (Lau et al., 2018). It is reported that there are significant differences in the classification of intestinal flora in patients with chronic kidney disease at the level of phylum, genus and species (Li et al., 2019). Conversely, increasing urea concentration during CKD can accelerate the process of renal injury, leading to changes in the intestinal microbiota, thereby increasing the production of enterotoxins and changing the intestinal epithelial barrier (Feng et al., 2019). However, due to the influence of age, diet, region, race and other factors, there are great inconsistencies in the changes of flora in different studies (Zhao et al., 2021). The relationship between gut microbiota imbalance and its corresponding interventions and chronic kidney disease is becoming a research focus of scholars (Steiger et al., 2022).

The analysis and summary of the structure of knowledge, the evolution of research centers and frontiers are important contributions of information science (IS) for researchers (Chen et al., 2012). There is still a lack of studies analyzing gut flora and kidney diseases using bibliometrics, and there are no related articles targeting the gastrointestinal microbiomes or kidney diseases in-depth (Wernimont et al., 2020). Given the strong association and potential clinical value of gut flora and kidney disease shown in recent studies, there is a need for systematic, intuitive, scientific bibliometric analysis and visualization of the growing number of original research articles linking the gastrointestinal microbiome and kidney disease.

CiteSpace is one of the representative bibliometric analysis applications based on citation analysis theory (Chen et al., 2012). It is used to extract important information of a certain domain, clarify the past and present development history and identify the most active research frontiers and trends of it (Chen et al., 2014). Here, we use CiteSpace to explore the research field of gastrointestinal microbiomes and kidney diseases to detect the contribution of the main countries, institutes, authors, journals and the progression of this domain.

## Methods

### Data collection

We used the terms “gastrointestinal microbiomes” and “kidney diseases” and all of their hyponyms in Medical Subject Headings (MeSHs) provided by the National Library of Medicine (NLM)/PubMed. We search Web of Science Core Collection (WoSCC) completely and loaded all literature in the field from 1990-2021 (the maximum analysis spectrum allowed by CiteSpace software), and the literature type was set as “articles”.

**Figure 1:**
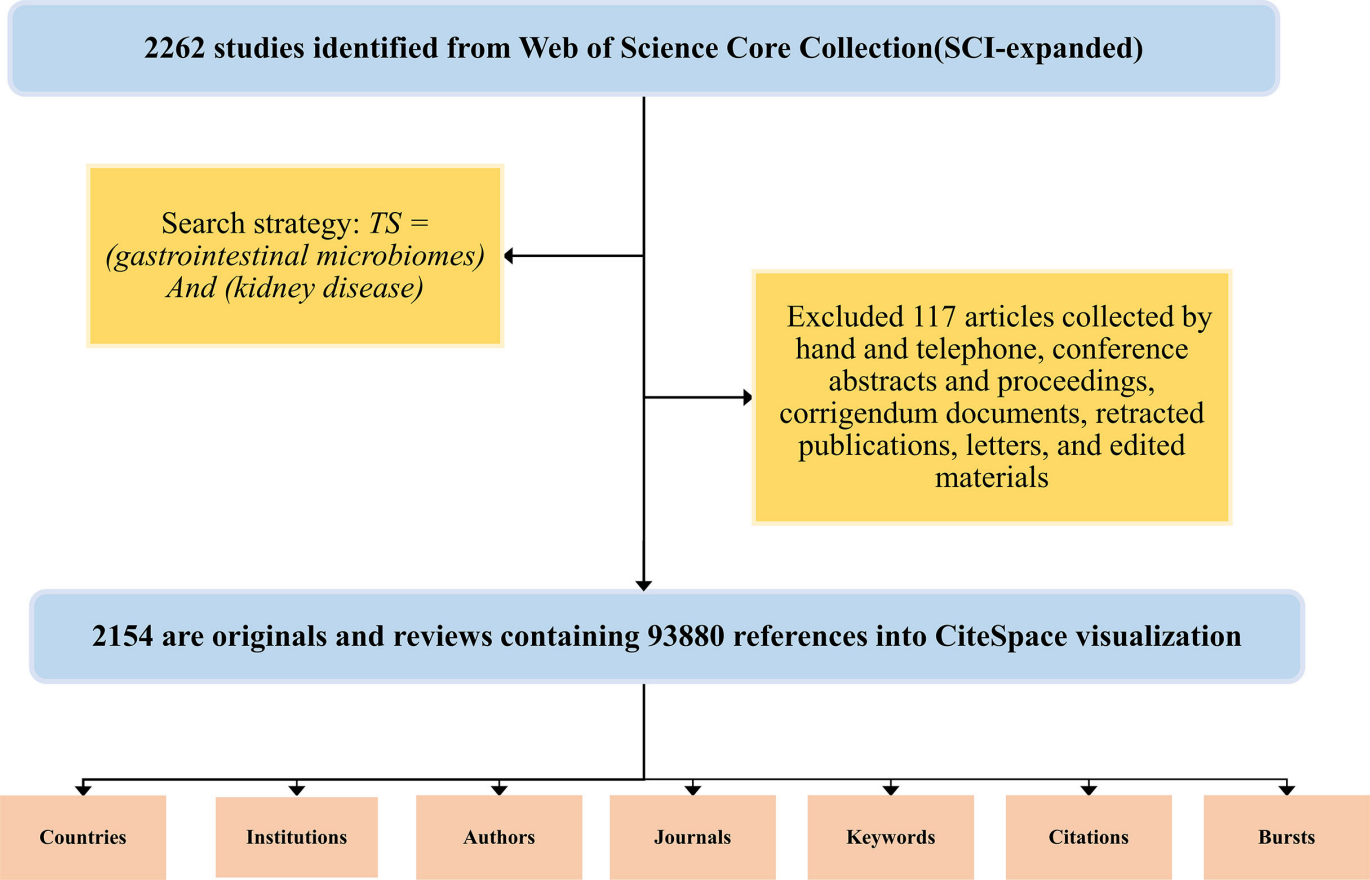
Workflow diagram of the publications search, screening, and analysis.

CiteSpace is a Java-based data analysis and document visualization application with outstanding functions, including co-citations, co-authors, and co-occurring keyword analyses, which helps to provide instant directions for analysis progression in a certain research field (Chen, 2004). Three core concepts have unique characteristics in CiteSpace analysis: burst detection, intermediate centrality, and heterogeneous network contributing to settling three practical concerns: research frontiers, remarkable keywords, and researching emerging trends (Chen et al., 2012; Chen et al., 2014). The exclusion criteria were: articles collected by hand and telephone; conference abstracts and proceedings, corrigendum documents; retracted publication; letters; edited materials. The search results were downloaded on August 10, 2021, and final 2145 valid results were recorded to CiteSpace for further analyses. In this article, we apply CiteSpace 5.8. R2 to analyze the countries, institutions, authors, journals, key clusters, citation relationships, and emerging trends in references (**Figure 1**).

## Results

### Distribution of articles by publication year

According to the Web of Science database, we retrieved 2262 studies among which 2154 are originals and reviews containing 93880 references. In 102 journals, 899 keywords were extracted by 973 main authors from 110 countries.

The earliest article in the field of gastrointestinal microbiomes and kidney diseases was published in English by Ramosalvarez, M, who used monkey kidney tissue to cultivate the intestinal flora collected from healthy children to determine whether there were poliovirus or other viruses in the children’s intestines (Ramos-Alvarez and Sabin, 1956).

In the next two decades, the research in the field of gastrointestinal microbiomes and kidney diseases is relatively scattered and marginal in the preliminary exploration without a representative research cluster, and in-depth analyses of the specific relationship between gastrointestinal microbiomes and kidney diseases were ignored, with only 6 articles published from 1956 to 1989. It was not until 1990 that the number of articles in this field began to accumulate steadily, surging from 5 to 2139 manuscripts exponentially.

### Co-country analysis

**Figure 2:**
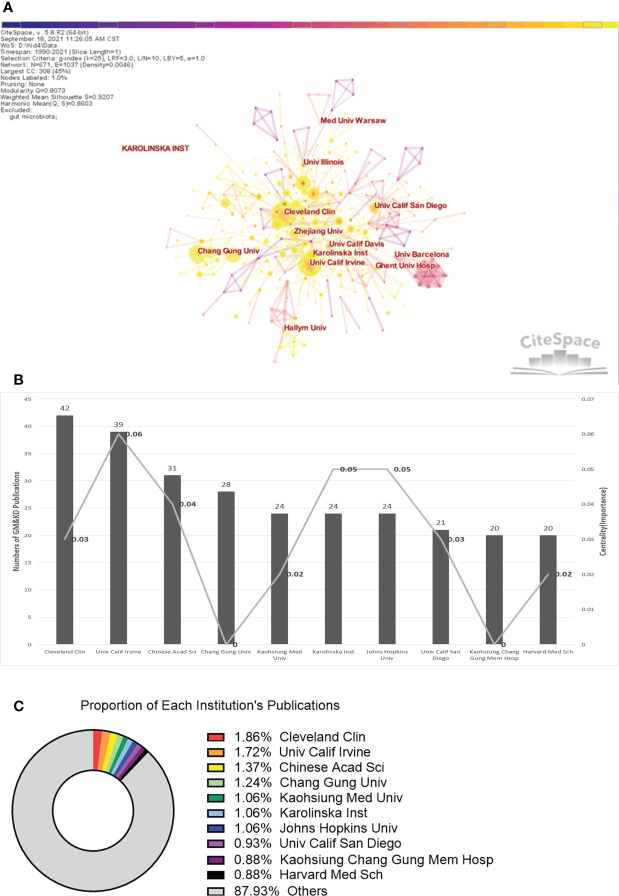
Co-institution knowledge map in the field of gastrointestinal microbiome and kidney disease during 1990-2021. **(A)** Nodes show in the form of annual rings that the annual ring width represents how many papers the institution publishes in a given year. The more papers are published, the wider the ring is in that year. And the brighter the node is, the newer the publication. **(B)** Total distribution comparison of the top ten most published countries and centralities in the field of gastrointestinal microbiomes and kidney diseases. **(C)** Proportion of the top ten most published institutions.

Co-country analysis by the CiteSpace algorithm can reveal the regionality of the research cooperation net in this field, which is displayed in **Figure 2**. The circle range represents the number of published studies attributed to the country in the **Figure 2A**. The most significant node in the center is the United States, which has the earliest exploration in this field of research and the largest number of works. It is immediately apparent in **Figure 1A** that many countries tend to collaborate with a relatively stable group of collaborators, generating clump-like research clusters.

**Figure 2B** is a column chart showing the number and centrality of publications in the first ten active countries in the field of gastrointestinal microbiomes and kidney diseases. The United States has 664 publications with a centrality of 0.45, followed by China with 489 and 0.11. The following countries are Italy, Japan, and Germany, of which the total numbers of publications are relatively close. The proportions of the top ten national productions in the field of gastrointestinal microbiomes and kidney diseases are presented in **Figure 2C**. The overall number of publications in the top ten countries, filled with bright color in the chart, occupies nearly 90% of total publications, which further illustrates that the research in this field has a high degree of regional concentration.

**Figure 2D** is a line chart showing the number of annual publications in the three countries with the largest volume, sorted as the United States, China, and Italy. The number of annual publications in the United States began to grow rapidly in 2011, while that of China and Italy were slightly later, increasing significantly in 2014. Although the number of the United States is relatively large, China’s growth rate is even greater, present in a steeper exponential function pattern, and even surpassing the United States in the number of articles published in 2020.

### Co-institution

**Figure 3:**
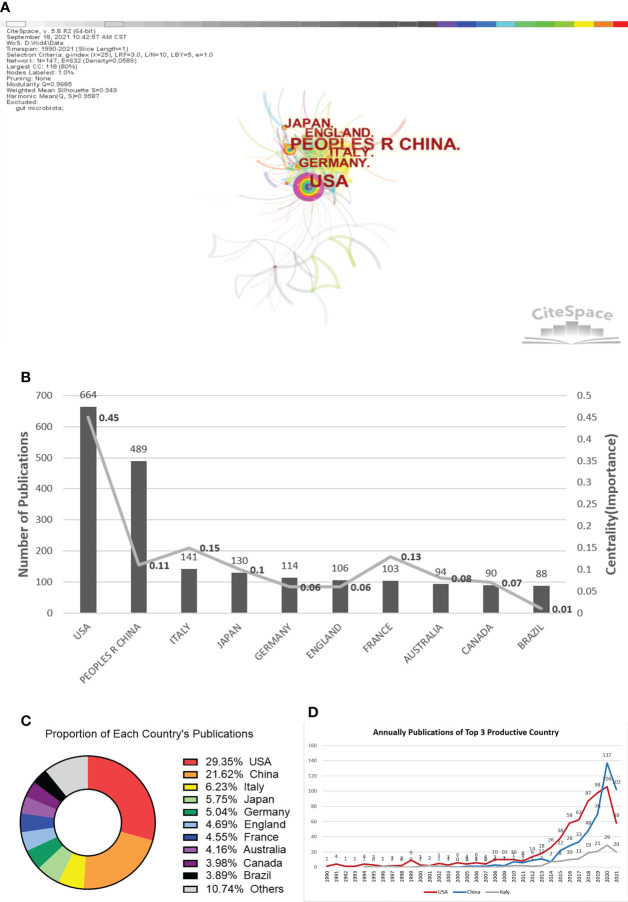
Co-country analysis in the field of gastrointestinal microbiomes and kidney diseases during 1990-2021. **(A)** Nodes show in the form of annual rings that the annual ring width represents how many papers the country publishes in a given year and the color. Nodes marked with purple circles represent convincible betweenness centrality, which stands for the importance of a certain object, and the warmer color the node is, the newer the publish is. The more papers are published, the wider the ring is in that year. **(B)** Total distribution comparison of the top ten most published countries and centralities in the field of gastrointestinal microbiomes and kidney diseases. **(C)** Proportion of the top ten most published countries. **(D)** Annual publications of the top three productive countries.

CiteSpace software mainly judges cooperation between two institutions when their names appear in one publication through the co-occurrence frequency matrix. **Figure 3** portrays the cooperative institutions in the field of gastrointestinal microbiomes and kidney diseases. In **Figure 3A**, the size of the node circle represents the number of papers published by the institute. The shorter the distance between the two circles, the greater the cooperation between the two institutions. Clusters betweenness centrality by outlining nodes with purple, which represent the importance. The cooperation between multiple institutions appears close, and the cutting-edge research is concentrated in Cleveland Clinic (42), the University of California, Irvine (39), and the Chinese Academic of Sciences in China (31). **Figure 3B** shows the statistics of the number and betweenness centrality of the top ten institutions by publication volume. It can be seen that the research in this field is centered at the University of California, Irvine (0.06), Karolinska Institutet (0.05), and Johns Hopkins University (0.05), whose betweenness is higher than that of other institutions. **Figure 3C** shows the proportion of all publications in this field occupied by the top 10 institutions. It can be seen intuitively that research in this field is highly concentrated in Cleveland Clinic (1.86%), the University of California, Irvine (1.72%), and the Chinese Academic of Sciences in China (1.37%).

### Co-authorship and journal analysis

We list the top ten authors with the highest productivity in this field and conclude their key contributions in this field (**Table 1**). Aiming at the exploration of the cooperative network in this specific research field, the size of a node representing the count of the corresponding article has been cited in the dataset.

**Table 1:** Top 10 most productive authors in the field of gastrointestinal microbiomes and kidney diseases.

Rank	Author	Count	Year	Representative article in this field
1	Denise Mafra	32	2015	Probiotics and chronic kidney disease
2	Nosratola D Vaziri	30	2012	Chronic kidney disease alters intestinal microbial flora
3	Denis Fouque	23	2013	Probiotics and chronic kidney disease
4	Youlin Tain	19	2018	Maternal Administration of Probiotic or Prebiotic Prevents Male Adult Rat Offspring against Developmental Programming of Hypertension Induced by High Fructose Consumption in Pregnancy and Lactation
5	CHIENNING HSU	18	2018	Maternal Administration of Probiotic or Prebiotic Prevents Male Adult Rat Offspring against Developmental Programming of Hypertension Induced by High Fructose Consumption in Pregnancy and Lactation
6	MarkusmHeimesaat	16	2012	Intestinal Microbiota Shifts towards Elevated Commensal Escherichia coli Loads Abrogate Colonization Resistance against Campylobacter jejuni in Mice
7	Stefan Bereswill	15	2012	Intestinal Microbiota Shifts towards Elevated Commensal Escherichia coli Loads Abrogate Colonization Resistance against Campylobacter jejuni in Mice
8	Loreto Gesualdo	15	2014	Microbiota and Metabolome Associated with Immunoglobulin A Nephropathy (IgAN)
9	W.H.Wilson Tang	14	2015	Gut Microbiota-Dependent Trimethylamine N-Oxide (TMAO) Pathway Contributes to Both Development of Renal Insufficiency and Mortality Risk in Chronic Kidney Disease
10	Jennifer L Pluznick	12	2013	Olfactory receptor responding to gut microbiota-derived signals plays a role in renin secretion and blood pressure regulation

The most productive authors in the research field of gastrointestinal microbiomes and kidney diseases is Denise Mafra, with 32 articles appearing in more than one article’s citation, focusing on renal nutrition and inflammation in CKD patients (Koppe et al., 2015). The second is Nosratola D. Vaziri whose contributions are focused on the effects of dietary and pharmacological interventions on the microbiome in uremia and/or chronic kidney disease (Vaziri et al., 2013). The following is Denis Fouque, studying nutrition and metabolism in kidney diseases and evidence-based nephrology (Koppe et al., 2015).

All articles in the research field of gastrointestinal microbiomes and kidney diseases have been published in a total of 1,105 journals. We identified the top 10 most productive journals in this field (**Table 2**). Plos One ranks first in the number of publications (1195), followed by Nature (1032) and Kidney International (956). All of this can provide important submission information for researchers.

**Table 2:** Top 10 most productive journals in the field of gastrointestinal microbiomes and kidney diseases.

Journals	Impact Factors (2021)	Number of publishedpapers
PLoS One	3.24	1195
Nature	49.962	1032
Kidney International	10.612	956
Journal Of The American Society Of Nephrology	10.121	857
Proceedings of the National Academy of Sciences of the United States of America	11.205	847
Science	47.728	743
Gut	23.059	721
New England Journal Of Medicine	91.245	713
Nephrology Dialysis Transplantation	5.992	712
Scientific Reports-UK	4.379	663

### Co-occurring keyword analysis

Co-cited keyword analysis offers the focus, research trends, and knowledge structure in this research field, extracting the topics that scholars in this field pay attention to. The analysis functions on word frequency, trend, burstiness and network attributes. **Table 3** shows the co-keyword burst detection results from 2011 to 2021. It can be seen that the keywords with an outbreak intensity (strength) greater than 10 are chronic renal failure (begin in 2013), carnitine (begin in 2015), and cardiovascular disease (begin in 2017). The greater the value of the outbreak intensity, the more emphasis the keyword is attached within this field. On the time axis column (1990-2021), the blue band represents the overall research duration in the field of intestinal flora and kidney disease, and the red cover represents the actual active duration of the keyword in this field. The longer the red band is, the longer the duration of this keyword. A recent important keyword is ***butyrate***, with a strength of 5.31, active from 2019 and continuing until 2021.

**Table 3:** Burst detection of Co-keyword analysis [Prone: MST g=10].

Keyword	Strength	Begin	End	1990-2021
chronic renal failure	10.05	**2013**	2017	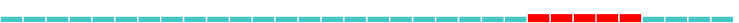
hemodialysis patient	7.96	**2013**	2017	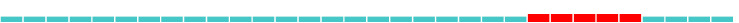
protein coupled receptor	7.03	**2014**	2016	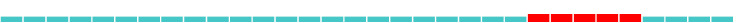
pathogenesis	6.07	**2014**	2016	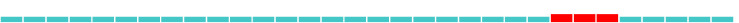
inflammatory bowel disease	5.63	**2014**	2018	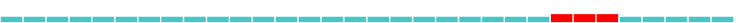
L-Carnitin	10.18	**2015**	2018	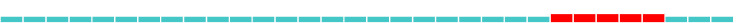
bound uremic toxin	7.21	**2016**	2018
atherosclerosis	5.8	**2016**	2018	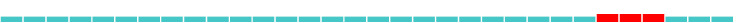
cardiovascular disease	10.39	**2017**	2018	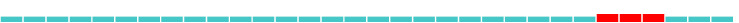
therapy	5.15	**2018**	2019	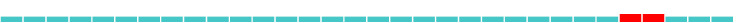
butyrate	5.32	**2019**	2021	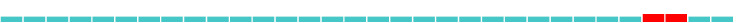

The whole length of the green bar represents the research topic in a time span of 31 years according to the CiteSpace setting, and the red bar represents the lasting time of the hotspot and corresponding timespan.

### Document co-citation analysis

**Figure 4:**
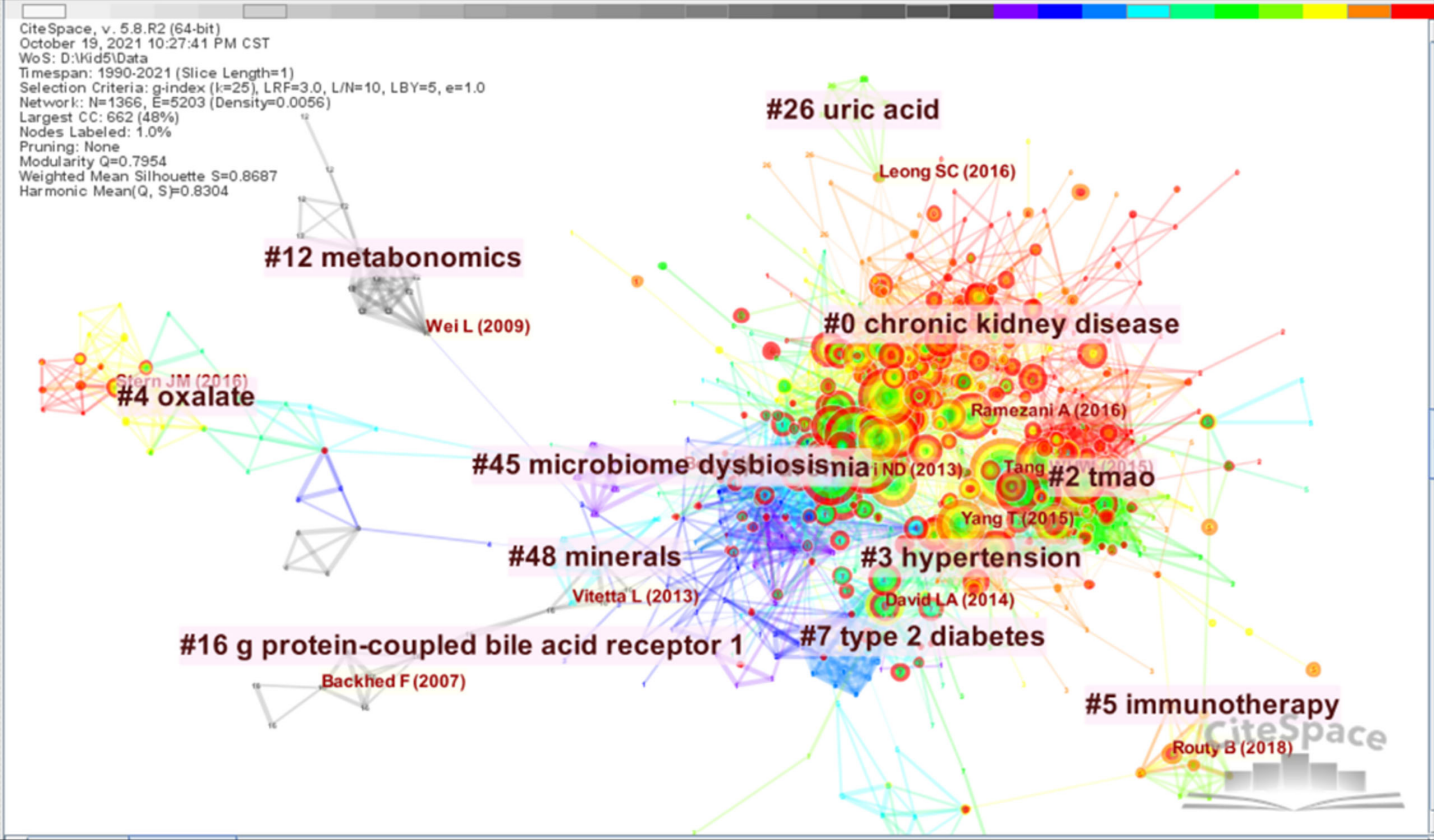
Co-citation knowledge map in the field of gastrointestinal microbiomes and kidney diseases during 1990-2021. Each node represents a research cluster, the size of the node represents the frequency of their occurrence, the line between nodes represents the degree of relevance, the thickness of lines indicates the strength of cooperation. The color of the lines and nodes corresponds to the publication time and the warmer color the color is, the newer the publish is.

A document co-citation network map is displayed in **Figure 4**, which contains 1366 independent nodes, 5203 lines, and 239 main clusters. The modularity Q is 0.7954, and the average value is 0.8687. As shown in **Figure 4**, “chronic kidney disease” is the largest cluster (#0), including 183 members, and has a mean publication year of 2016. The most frequently cited article is “Role of the Gut Microbiome in Uremia: A Potential Therapeutic Target” (Ramezani et al., 2016). This article reflects the researchers’ main interest in cluster #0. The second-largest cluster in this field of knowledge (ranked #1), “uremia”, has 149 member articles, and the average publication year is 2012. The most active citer is Vaziri, Nosratola D et al., 2013, attracting people’s attention to the variation between the intestinal flora in the gastrointestinal tract of people with and without kidney diseases and could be regarded as foundation work inspiring follow-up research on the specific mechanisms in this field (Vaziri et al., 2013).

The third-largest cluster is “trimetlylamine oxide (TMAO)”(ranked #2), consisting of 101 members and having a mean year of publication in 2016. TMAO is a typical uremic toxin produced by the intestinal flora’s metabolism, which means that research into the mechanism of kidney disease has been profound (Tang et al., 2015; Agus et al., 2021). The most active citation is “Gut microbiota-dependent trimethylamine N-oxide pathway contributes to both development of renal insufficiency and mortality risk in chronic kidney disease” (Tang et al., 2015). The results of their study draw people’s attention to the metabolites of intestinal flora and the pathogenesis of cardiovascular disease, which broadens the frontiers of research in this field.

There are many burst-edge nodes of the organ and red color on the right side of **Figure 4**, suggesting the sharp increase in the number of citations of this document in a relatively short period and indicating the importance and innovation of this study. It shows that the literature published in this field naturally tends to have a high degree of popularity, and many researchers are working on exploring the derivative research of intestinal flora in the field of kidney diseases.

The co-citation analysis of gastrointestinal microbiomes and kidney diseases research produced 239 co-citation clusters marked with the index words in the respective citations. To describe the nature of this clustering, CiteSpace extracts noun phrases from article titles that cite clusters based on three specialized metrics: latent semantic indexing, log-likelihood ratio, and mutual information. The log-likelihood ratio usually provides the best results in terms of uniqueness and coverage of topics related to the cluster. **Table 4** summarizes the details of the 12 main clusters, all of which have contour values greater than 0.7, indicating reliable and meaningful results. In short, it can be concluded from the active citers and extracted words that the influence of the metabolites of the intestinal flora on the various systems within the disease state and the specific mechanism of action are the hotspots and advancements of current research.

**Table 4:** The largest 12 clusters of gastrointestinal microbiomes and kidney diseases document co-citation, identified by keyword.

Cluster ID	Size	Silhouette	Year	Label (LSI)	Label (LLR)	Label (MI)
0	183	0.783	2016	chronic kidney disease	chronic kidney disease	infrequent dialysis
1	149	0.833	2012	chronic kidney disease	uremia	minerals
2	101	0.967	2016	trimethylamine n-oxide	Trimetlylamine oxide	mortality prediction
3	89	0.809	2014	gut microbiota	hypertension	sensory receptor
4	37	1	2013	kidney stones	oxalate	tea
5	31	0.964	2014	renal cell carcinoma	immunotherapy	ascites
7	26	0.976	2012	chronic kidney disease	type 2 diabetes	antimicrobial
12	14	1	2006	compound list	metabonomics	chronic kidney disease
16	12	0.993	2007	farnesoid x receptor	G protein-coupled bile acid receptor 1	chronic kidney disease
26	9	0.996	2016	uremic toxins	uric acid	chronic kidney disease
45	6	0.995	2008	bacterial translocation	microbiome dysbiosis	chronic kidney disease
48	5	1	2011	…	minerals	chronic kidney disease

### Emerging trends and research focus

The tendency of research interests is highlighted through citation bursts with the measure of the innovativeness of research results and a frontier “footprint” of this study field (Chen et al., 2014). The larger the burst value is, the more innovative and important the research results are.

**Table 5** shows the top 25 references among a total of 170 references with the strongest citation bursts during the period between 1990 and 2021. The earliest four references highlight the emerging trend of gastrointestinal microbiomes and kidney diseases bursts in 2012, and studies have focused on exploring the mechanism by which the gut microbiota affects the human body through metabolites (Ranganathan et al., 2010; Meijers et al., 2010; Wang et al., 2011; Aronov et al., 2011). Wang, Zeneng et al. (2012) discovered the relationship between the gut microbiota-dependent metabolism of dietary phosphatidylcholine and the pathogenesis of cardiovascular disease (Wang et al., 2011).

**Table 5:** References with the strongest 25 citation bursts in the field of gastrointestinal microbiomes and kidney diseases.

References	Year	Strength	Begin	End	1990 - 2021
Wang ZN, 2011, NATURE, V472, P57, DOI 10.1038/nature09922, DOI	2011	21.31	2012	2016	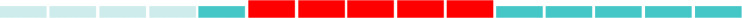
Aronov PA, 2011, J AM SOC NEPHROL, V22, P1769, DOI 10.1681/ASN.2010121220, DOI	2011	19.78	2012	2016	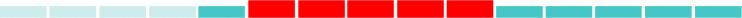
Ranganathan N, 2010, ADV THER, V27, P634	2010	13.05	2012	2015	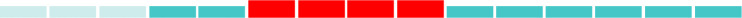
Meijers BKI, 2010, NEPHROL DIAL TRANSPL, V25, P219, DOI 10.1093/ndt/gfp414, DOI	2010	11.86	2012	2015	
Vaziri ND, 2013, KIDNEY INT, V83, P308, DOI 10.1038/ki.2012.345, DOI	2013	37.19	2013	2018	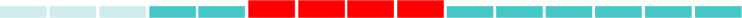
Anders HJ, 2013, KIDNEY INT, V83, P1010, DOI 10.1038/ki.2012.440, DOI	2013	16.61	2013	2018	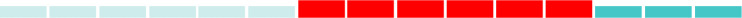
Vaziri ND, 2012, NEPHROL DIAL TRANSPL, V27, P2686, DOI 10.1093/ndt/gfr624, DOI	2012	14.58	2013	2017	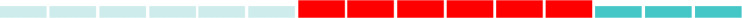
Vaziri ND, 2012, CURR OPIN NEPHROL HY, V21, P587, DOI 10.1097/MNH.0b013e328358c8d5, DOI	2012	12.98	2013	2016	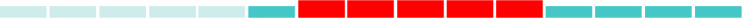
Nakabayashi I, 2011, NEPHROL DIAL TRANSPL, V26, P1094, DOI 10.1093/ndt/gfq624, DOI	2011	12.34	2013	2016	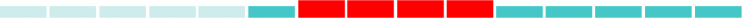
Vaziri ND, 2012, AM J NEPHROL, V36, P438, DOI 10.1159/000343886, DOI	2012	10.45	2013	2017	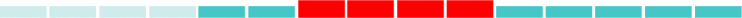
Koeth RA, 2013, NAT MED, V19, P576, DOI 10.1038/nm.3145, DOI	2013	22.91	2014	2018	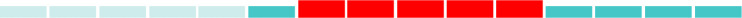
Pluznick JL, 2013, P NATL ACAD SCI USA, V110, P4410, DOI 10.1073/pnas.1215927110, DOI	2013	13.94	2014	2018	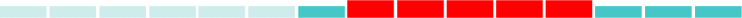
Vaziri ND, 2013, AM J NEPHROL, V37, P1, DOI 10.1159/000345969, DOI	2013	13.43	2014	2018	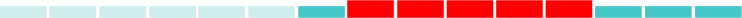
Qin JJ, 2012, NATURE, V490, P55, DOI 10.1038/nature11450, DOI	2012	12.87	2014	2017	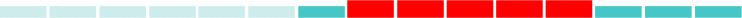
Wu IW, 2011, NEPHROL DIAL TRANSPL, V26, P938, DOI 10.1093/ndt/gfq580, DOI	2011	12.73	2014	2016	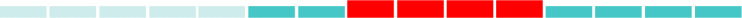
Wang FQ, 2012, NEPHROLOGY, V17, P733, DOI 10.1111/j.1440-1797.2012.01647.x, DOI	2012	12.48	2014	2017	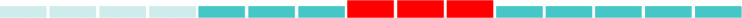
Tremaroli V, 2012, NATURE, V489, P242, DOI 10.1038/nature11552, DOI	2012	9.74	2014	2017	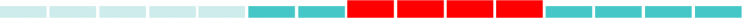
Tang WHW, 2013, NEW ENGL J MED, V368, P1575, DOI 10.1056/NEJMoa1109400, DOI	2013	20.35	2015	2018	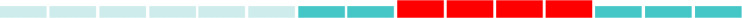
Wong J, 2014, AM J NEPHROL, V39, P230, DOI 10.1159/000360010, DOI	2014	15.41	2015	2019	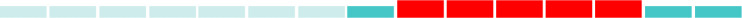
Ramezani A, 2014, J AM SOC NEPHROL, V25, P657, DOI 10.1681/ASN.2013080905, DOI	2014	12.95	2015	2019	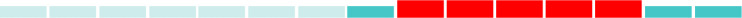
Vaziri ND, 2014, PLOS ONE, V9, P0, DOI 10.1371/journal.pone.0114881, DOI	2014	9.68	2016	2019	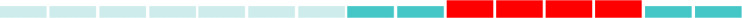
Sirich TL, 2014, CLIN J AM SOC NEPHRO, V9, P1603, DOI 10.2215/CJN.00490114, DOI	2014	9.68	2016	2019	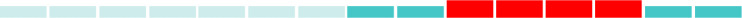
Li J, 2017, MICROBIOME, V5, P0, DOI 10.1186/s40168-016-0222-x, DOI	2017	10.46	2019	2021	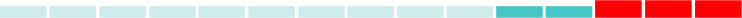
Lau WL, 2018, CLIN SCI, V132, P509, DOI 10.1042/CS20171107, DOI	2018	9.51	2019	2021	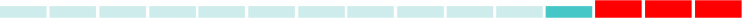
Wilck N, 2017, NATURE, V551, P585, DOI 10.1038/nature24628, DOI	2017	9.28	2019	2021	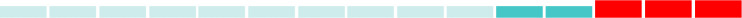

The whole length of the green bar represents the research topic in a time span of 31 years according to the CiteSpace setting, and the red bar represents the lasting time of the hotspot and corresponding timespan.

The following six references highlight the emerging trend that began in 2013. The main content of these studies is the changes in the intestinal flora of patients in disease states and their role in disease progression (Nakabayashi et al., 2011; Vaziri et al., 2012; Vaziri et al., 2012; Vaziri, 2012; Vaziri et al., 2013; Anders et al., 2013). The last three references received great attention in 2019 and continued until 2021, pointing out the current hotspot of gastrointestinal microbiomes and kidney diseases research, and the detailed mechanisms of the gut microbiome in metabolic diseases are further revealed (Wilck et al., 2017; Li et al., 2017; Lau et al., 2018). Looking into the track of these burst detections can reflect the developmental history of the gastrointestinal microbiomes and kidney diseases field (Chen et al., 2012).

**Table 6** summarizes the top five references with the highest citation counts in gastrointestinal microbiomes and kidney diseases. The top-ranked item in co-citation counts is Tang, W H Wilson et al. (2015) in Cluster [1] with a citation count of 159 (Tang et al., 2015), followed by Vaziri ND et al. (2013) in Cluster [2] with a citation count of 155 (Vaziri et al., 2013), Ramezani A et al. (2014) in Cluster [1] with a citation count of 133 (Ramezani et al., 2016), and Wong J et al. (2014) in Cluster [2] with a citation count of 130 (Wong et al., 2014). The fifth is Rossi M et al. (2016) in Cluster [1], with a citation count of 102 (Rossi et al., 2016).

**Table 6:** References with the strongest citation frequency in gastrointestinal microbiomes and kidney diseases. *(TMAO: Trimetlylamine oxide)*.

Frequency	Burst	Degree	Centrality	Sigma	Author	Year	Title	Source	Cluster
159		28	0.01	1	Tang WHW	2015	***Gut microbiota-dependent trimethylamine N-oxide (TMAO) pathway contributes to both development of renal insufficiency and mortality risk in chronic kidney disease***	Circulation Research	#2 TMAO
155	37.19	39	0.01	1.41	Vaziri ND	2013	***Chronic kidney disease alters intestinal microbial flora***	Kidney International	#1 uremia
133	12.95	15	0.01	1.07	Ramezani A	2014	***The gut microbiome, kidney disease, and targeted interventions***	Journal of The American Society of Nephrology	#1 uremia
130	15.41	45	0.02	1.39	Wong J	2014	***Expansion of urease- and uricase-containing, indole- and p-cresol-forming and contraction of short-chain fatty acid-producing intestinal microbiota in ESRD***	American Journal of Nephrology	#1 uremia
102		50	0.03	1	Rossi M	2016	***Synbiotics Easing Renal Failure by Improving Gut Microbiology (SYNERGY): A Randomized Trial***	Clinical Journal of the American Society of Nephrology	#1 uremia

Tang, W H Wilson et al. study the clinical prognostic value of TMAO in chronic kidney disease (CKD) patients and non-CKD patients and to verify the hypothesis that TMAO plays a direct role in the occurrence and development of renal dysfunction. They found that elevated plasma TMAO levels in patients with CKD predicted poor long-term survival (Tang et al., 2015).

Vaziri ND et al. proves that diet and drug intervention for uremia and/or chronic kidney disease would profoundly change the intestinal microbiome, though the specific mechanism was remained unclear at that time (Vaziri et al., 2013). Ramezani A et al. reviewed the quantitative and qualitative changes in the intestinal flora of patients contribute to the progression and treatment of CKD (Ramezani and Raj, 2014).

Wong, Jakk et al. found that the bacterial family with urease, uricase, indole, and p-cresol forming enzyme in the End Stage Renal Disease patients significantly expand and contribute to the toxicity and inflammation of uremia in intestinal microbial metabolis (Wong et al., 2014).

Rossi, Megan et al. found that synbiotics do not significantly reduce serum is, but do reduce serum PCs, which is conducive to improving fecal microbiome in patients with CKD (Rossi et al., 2016).

## Discussion

The relationship between gut microbiota imbalance and its corresponding interventions and kidney disease is becoming a research focus of scholars (Ikee et al., 2020; Wang et al., 2020; Yin et al., 2021). Our study presents a quantitative bibliometric method and explores the progress in the domain of gastrointestinal microbiomes and kidney diseases. In this field, the proportions of China and the United States are far greater than those of other countries, demonstrating their great contribution to this study field. Research related to intestinal flora and kidney disease has attracted considerable attention around the world, but each region has different foci. The United States has the most active research institutions and the longest research history. One of the representatives is Cleveland Clinic, of which the most-cited article explored the relationship between the metabolites of the intestinal flora and cardiovascular disease (Tang et al., 2015). The University of California, Irvine also contributes greatly to research in this field, mainly based on Vaziri ND, focusing on the mechanisms of oxidative stress in a chronic kidney disease condition (Vaziri et al., 2012; Vaziri et al., 2012; Vaziri, 2012; Vaziri et al., 2013; Vaziri et al., 2013). Although China’s research into this field was once started somehow lagging out, the strong research potential in this research field cannot be underestimated (Li et al., 2017). The contribution of European countries is mainly associated with nutrition and focusing on renal nutrition and inflammation (Koppe et al., 2015).

Derived from the document co-citation cluster markers, it can be noticed that scholars and experts are interested in the influence of metabolic activities in the intestine and the subsequent reaction brought by its metabolic interaction on patients (Brown and Hazen, 2017). According to highly cited pieces of literature, the mechanism of action and regulatory means of metabolic products of intestinal flora on other systems interacting with the urinary system are hot spots and frontiers of research (Sircana et al., 2019). Immunotherapy, microbial metabolism, and cellular barriers have been studied in great depth and detail (Brown and Hazen, 2017; Li et al., 2017).

Burst detection and keyword visualizes the research hotspots intuitively. The core knowledge domains in gastrointestinal microbiomes and kidney diseases research are chronic renal failure, L-Carnitin, and cardiovascular disease. It could be speculated from the citation bursts that the pathogenesis of uremica toxin is an emerging trend in gastrointestinal microbiomes and kidney diseases. Probiotic or synbiotic supplements are being studied to adjust abnormal intestinal symbiotic environments, delaying or halting the progression of the disease (Zheng et al., 2021).

Synthesizing the evolution of citation clusters and burst detection, the footprint in the field of gastrointestinal microbiomes and kidney diseases is present clearly. Intestinal flora imbalance refers to abnormalities in the abundance, uniformity, and composition of microbial communities that are prevalent in chronic kidney disease, including a marked decrease in probiotics and a significant increase in potentially pathogenic bacteria (Rinninella et al., 2019; Mafra et al., 2019; Meijers et al., 2019). Gut dysbiosis plays an important role in abnormal intestinal metabolism, producing more uremic toxins, such as p-cresol sulfate, trimethylamine-N-oxide, and indole sulfate, which are closely related to chronic kidney disease. At the same time, the effects of these uremic toxins in chronic kidney disease patients with cardiovascular disease have gradually attracted attention (Li et al., 2017; Tang et al., 2019; Naghipour et al., 2021). Conversely, a reduction in the beneficial bacteria in the gut reduced their metabolites, short-chain fatty acids, which can improve the structure of intestinal flora, promote the reproduction of beneficial bacteria, and inhibit the growth of potentially pathogenic bacteria (Sircana et al., 2019; Swanson et al., 2020). A growing body of literature suggests that prebiotics, probiotics and synbiotic supplements can correct the imbalanced gut microbiota structure in patients with chronic kidney disease, improve gut microbial metabolism, reduce inflammation, inhibit oxidative stress, and regulate lipid metabolism, and other important functions are expected to become a new approach for the treatment of chronic kidney disease (Wu et al., 2017; Mazidi et al., 2017; Lopes et al., 2018).

The analysis of co-authors shows that researchers make outstanding contributions to the field. The earliest reference with a strong citation burst is Wang, Zeneng, et al., 2011. Their discovery of a relationship between gut flora-dependent metabolism of dietary phosphatidylcholine and cardiovascular disease pathogenesis provides opportunities for the development of new diagnostic tests and therapeutic approaches for atherosclerotic heart disease and can be regarded as the milestone finding that inspired subsequent research (Wang et al., 2011). Aronov PA et al. compared the plasma of patients with and without colon hemodialysis to identify and further characterize colon-derived uremic solutes, finding that colonic microbes may produce an important portion of uremic solutes (Aronov et al., 2011). Vaziri ND et al. attracted people’s attention to the variation between the intestinal flora in the gastrointestinal tract of people with and without kidney diseases and could be regarded as foundation work inspiring follow-up research on the specific mechanisms in this field (Vaziri et al., 2012; Vaziri et al., 2012; Vaziri, 2012; Vaziri et al., 2013). Koeth RA et al. demonstrated that the metabolism of the intestinal microbiota of dietary L-Carnitin (trimethylamine rich in red meat) also produces TMAO and accelerates atherosclerosis in mice (Koeth et al., 2013). They believe that the intestinal microbiota may contribute to high levels of red meat consumption and the risk of common cardiovascular disease. Wang ZN et al. conducted a large cohort study in 2011, focusing on the relationship between the intestinal flora-dependent metabolism of phosphatidylcholine in diet and cardiovascular pathogenesis. The discovery of the relationship between the intestinal flora-dependent metabolism of dietary phosphatidylcholine and the pathogenesis of cardiovascular disease provides an opportunity to develop new diagnostic tests and treatments for atherosclerotic heart disease (Wang et al., 2011; Tang et al., 2013).

There are some strengths of this study. The combination of knowledge maps and visual analysis could simultaneously reflect the current status of research quantitatively present the distribution of collaboration among countries, institutions and authors. Secondly, this study summarizes the key contribution and turning points in this fields. Third, this study reports the latest progress in the research field visual through co-cited reference analysis and keyword analysis. We admit this bibliometrics study has some inherent limitations. First, only WoSCC database was analyzed in this study due to the inherent algorithm of CiteSpace. Some articles published in other databases may be omitted. Second, the non-English article may be underestimated according to the evaluated system of CiteSpace.

## Conclusion

Over the past two decades, the scientific collaboration network showed that collaboration between different countries and institutions has been adequate, with a gradual expansion of research scope and depth over time. The core contributors in the field of research on intestinal flora and kidney disease are the United States and China. Research in recent years has focused on the effects of imbalanced gastrointestinal microbiome metabolites on various human systems and the clinical value provided by probiotic and synbiotic supplementation. The interaction between gastrointestinal microbiomes and kidney diseases is expected to be a promising perspective for the treatment of chronic kidney disease. However, these mechanisms have not yet been clearly investigated, and further studies are needed to corroborate them.

## Data availability statement

The original contributions presented in the study are included in the article/supplementary material. Further inquiries can be directed to the corresponding authors.

## Author contributions

WQ conceived the study and performed critical revision of the manuscript. JT designed the study, drafted the manuscript and supervised the study. MC designed the study, performed statistical analyses and wrote the manuscript. YW processed statistical data and developed the analyses. YT performed the article retrieval and data interpretation. All authors read and approved the final manuscript.

## Funding

This work was supported by National Natural Science Foundation of China No. 81970612.

## Conflict of interest

The authors declare that the research was conducted in the absence of any commercial or financial relationships that could be construed as a potential conflict of interest.

## Publisher’s note

All claims expressed in this article are solely those of the authors and do not necessarily represent those of their affiliated organizations, or those of the publisher, the editors and the reviewers. Any product that may be evaluated in this article, or claim that may be made by its manufacturer, is not guaranteed or endorsed by the publisher.
